# Effect of Aging-Induced Dioxolane Polymerization on the Electrochemistry of Carbon-Coated Lithium Sulfide

**DOI:** 10.3389/fchem.2019.00893

**Published:** 2020-01-10

**Authors:** Lucas Lodovico, Alberto Varzi, Stefano Passerini

**Affiliations:** ^1^Helmholtz Institute Ulm (HIU), Ulm, Germany; ^2^Karlsruhe Institute of Technology (KIT), Karlsruhe, Germany

**Keywords:** lithium-sulfur battery, lithium sulfide, dioxalane, electrolyte aging, N-rich carbon-coating, ethylenediamine

## Abstract

Lithium sulfide-based materials have been considered as potential positive electrodes for the next generation batteries. Lithium sulfide is the fully lithiated form of sulfur, i.e., they share the same high theoretical capacity. However, it has the benefit of already containing lithium, which allows making cells with lithium-free negative electrodes. Lithium sulfide, however, shares with sulfur the polysulfide dissolution drawback upon cycling. One possible solution to this problem is to envelop the active material particles with carbonaceous materials. In this work, we investigate the effect of a nitrogen-rich carbon coating on lithium sulfide particles. The effect of such coating on the surface properties and electrochemistry of lithium sulfide cathodes is investigated in details, in particular, regarding its interaction with fresh vs. aged electrolyte. The polymerization of dioxalane (DOL) due to aging is found to affect the electrochemistry of lithium sulfide and, interestingly, to improve the cycling performance.

## Introduction

Lithium-sulfur batteries (LSBs) have attracted much attention in recent years, with the prospect of replacing lithium-ion batteries (LIBs) as energy source for automotive and, especially, flight applications as soon as 2030 (International and Agency, 2018). The main reason for this is the high theoretical capacity of sulfur, around 1,675 mAh g^−1^, promising Li-S batteries with energy densities (based on the electrode materials only) as high as 2,600 Wh kg^−1^. Sulfur-based cathodes, however, still possess a multitude of problems that need to be solved. The main challenge posed by sulfur is the high solubility of the intermediates formed during cycling (Xu et al., 2015). These intermediates take the form of polysulfides with general formula Li_2_S_x_, which are chain of sulfur atoms with x usually comprised between 3 and 8. At the end of discharge, solid discharge products (Li_2_S and Li_2_S_2_) are finally formed. The first and most obvious consequences of polysulfides dissolution are low Coulombic efficiencies and loss of active material during battery operation.

In order to avoid these effects, a commonly used strategy is to physically constrain the polysulfides in the positive electrode (cathode), often by enveloping the active material's particles with an inert coating, such as carbon (Jeong et al., 2013; Agostini et al., 2014; Chen et al., 2014; Nan et al., 2014; Hwa et al., 2015; Wu et al., 2016). Carbon coating of sulfur, however, is all but trivial, owing to its low melting point and tendency to easily sublimate at moderate temperature. In these terms, the use of lithium sulfide as cathode active material presents interesting advantages. Besides being already in the lithiated state, which allows it to be paired with lithium-free negative (anode) electrodes (Nanda et al., 2018), lithium sulfide is thermally stable up to rather high temperature allowing processing such as carbonization.

In this work, we investigate the effect of a nitrogen-rich carbon coating on lithium sulfide particles. Specifically, ethylenediamine-embedded lithium sulfide, previously reported by our group (Lodovico et al., 2019), is pyrolized in order to obtain carbon-coated lithium sulfide, with the ethylenediamine molecules serving as the carbon source. The use of this kind of diamine lead to a N-doped carbon coating with C:N weight ratios around 2:1. The effect of such coating on the surface properties and electrochemistry of lithium sulfide cathodes is investigated in details, in particular, regarding its interaction with fresh vs. aged electrolyte. It was found that upon prolonged storage (12+ months) the dioxolane-based electrolyte tends to polymerize, which greatly affects the behavior of Li_2_S-based cathodes.

## Methods

Chemical and electrode preparations, infrared measurements, and cell assembly were carried out in a glove box (MBraun) filled with argon (O_2_ and H_2_O content below 0.1 ppm). The 1:1 volume ratio mixture of Dimethoxyethane (DME, Solvionic) and 1,3-Dioxolane (DOL, Solvionic) was used as the electrolyte solvent. Prior to mixing, the two solvents were individually dried using 3Å molecular sieves (Sigma Aldrich) to reach water contents below 20 ppm as determined via Karl-Fischer titration (Mettler-Toledo Titrator Compact C30). Appropriate amounts of lithium bis(trifluoromethanesulfonyl)imide (LiTFSI, Solvionic) and lithium nitrate (LiNO_3_, Alfa Aesar) were dissolved into the DME-DOL solvent mixture to achieve concentration of 1 and 0.25 mol L^−1^, respectively, using aluminum bottles. This electrolyte was used soon after its preparation (i.e., within 2 weeks; labeled as “fresh”) or after storage (i.e., after more than 1 year, labeled “aged”). Besides the aging, both the electrolytes were stored in sealed aluminum bottle inside the glove box.

Carbon-coated lithium sulfide (Li_2_S-CC) was prepared using ethylenediamine-containing lithium sulfide (Li_2_S-En) as precursor. Li_2_S-En synthesis, carbon coating procedure, and electrode preparation is described in a previous work (Lodovico et al., 2019). The final material possesses a particle coating with a C:N weight ratio of about 2:1.

Li_2_S-CC was employed as active electrode materials. The electrodes were prepared by manually grind-mixing the active material (Li_2_S-CC) with the conductive carbon (Super C65; Imerys Graphite & Carbon) and the binder (polyvinylidene fluoride; PVdF 6020 from Solvay). The respective weight ratios were 53:37:10. The mixed powders were then dispersed with N-Methyl-2-pyrrolidone (NMP, Sigma Aldrich), and the resulting slurry cast over aluminum current collectors using the doctor blade method. Finally, the electrode tapes were dried at 60 °C under Ar.

For the determination of the electrolyte electrochemical stability window, carbon electrodes composed of Super C65 and PVdF were prepared in the 85:15 weight ratio. NMP was added to prepare the slurry, which was cast over the aluminum current collector as well.

Carbon electrodes were also prepared using nitrogen-rich carbon derived from carbonization of 1-ethyl-3-methylimidazolium tricyanomethanide (EMImTCM, IoLiTec). In short, EMImTCM was placed in an alumina boat inside a horizontal tube furnace under argon flow. The temperature was raised to 500°C at a rate of 5°C min^−1^ and kept for 5 h. The resulting carbonized sample (N-rich carbon) was composed of 62% carbon, 33% nitrogen, 2% hydrogen, and 3% oxygen (all values given as weight percentages). This composition is rather similar to that of the carbon coating in Li_2_S-CC. The N-rich carbon was used to prepare electrodes as well, in which Super C-65, N-rich carbon, and PVdF were mixed in a 70:15:15 weight ratio, dispersed in NMP, and cast over aluminum foil, and dried at 120°C under vacuum for 48 h.

Electrochemical tests were performed using three electrode T-type cells (Swagelok), with lithium metal as both the reference and counter electrodes. The cells were assembled using 100 μL of electrolyte soaked into glass fiber separators (Whatman GF/A). For the capacity retention experiments, the cells stored at 20°C in climatic chamber (Binder) were galvanostatically cycled using a battery tester (Maccor S4000). The Li_2_S-CC based cells were activated by charge at a C/20 rate (1C = 1,165 mAh g^−1^) up to 4 V vs. Li/Li^+^, followed by cycling at C/10 between 1.9 and 3.0 V vs. Li/Li^+^.

Electrochemical impedance spectroscopy (EIS) measurements were performed using a BioLogic VMP3 multi-channel potentiostat. For that, the cells were cycled using the same procedures as mentioned above. However, after each discharge, the cell was allowed to rest at open-circuit for 2 h prior to measuring impedance. Spectra were recorded between frequencies of 200 kHz and 100 mHz, using a 5 mV signal amplitude.

The anodic electrochemical stability of the electrolytes was studied by cyclic voltammetry (CV), where the potential was scanned from 3 to 4 V vs. Li/Li^+^. After cycling, each cell was disassembled inside a glove box, and the electrolyte recovered by centrifuging the glass fiber separators at 6,000 rpm for 10 min. The recovered electrolytes were analyzed via ATR-IR.

## Results and Discussion

**Figure 1:**
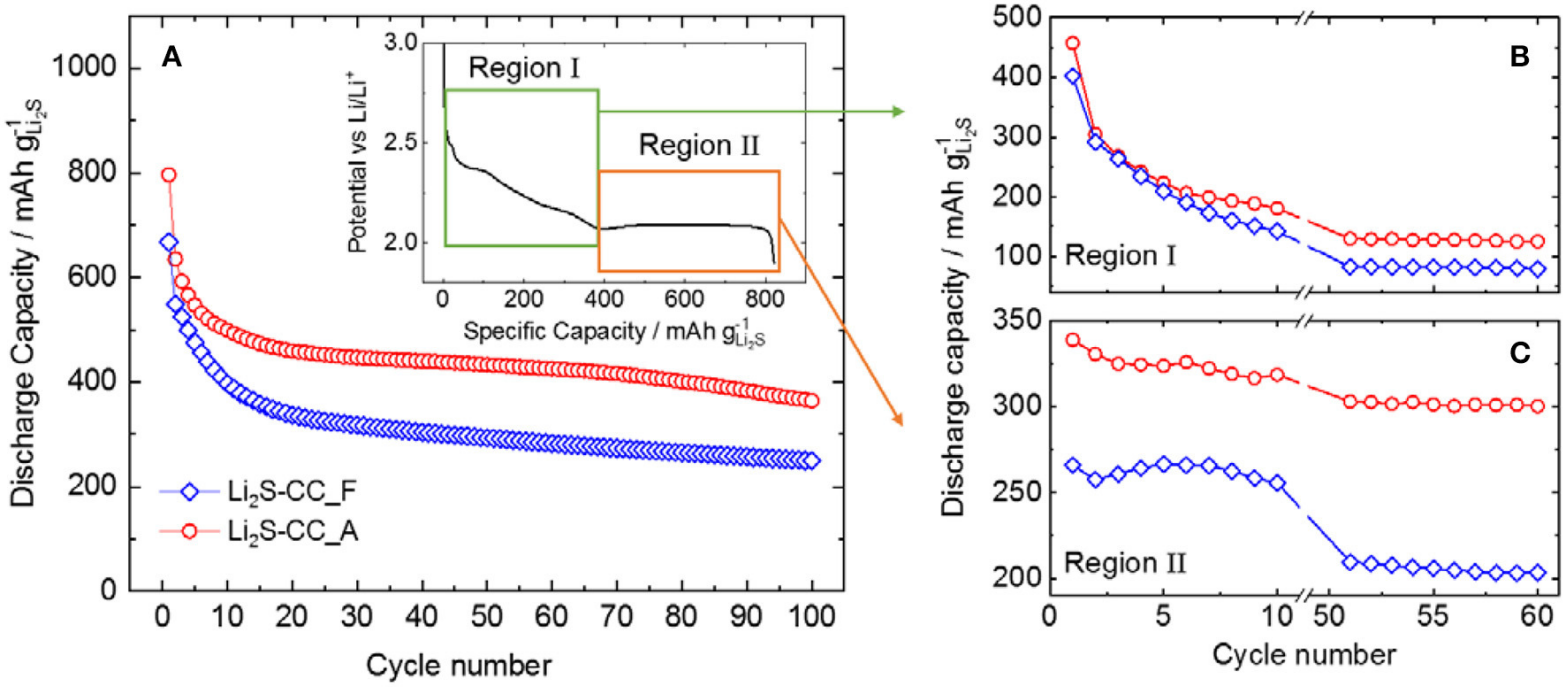
**(A)** Capacity retention for cells using carbon-coated Li_2_S either in fresh (Li_2_S-CC_F) or aged electrolyte (Li_2_S-CC_F). Inset: Typical discharge profile showing the two main discharge plateaus. Evolution of the capacity of **(B)** region I and **(C)** region II for selected cycles.

Figure 1A shows the capacity retention of Li_2_S-CC-based electrodes when using either the fresh (Li_2_S-CC_F) or the aged (Li_2_S-CC_A) electrolyte. It is evident that the electrolyte aging has a large impact on the cell performance. Surprisingly, the aged electrolyte provides higher discharge capacity as well as capacity retention upon 100 cycles. In order to better understand the reason behind this effect, the discharge curves of the cathode in each electrolyte were closely examined. The inset of Figure 1A shows the typical discharge curve of a Li_2_S-based electrode after the initial activation charge leading to fully oxidized S. The first region (I) shows a potential plateau (around 2.4 V vs. Li/Li^+^) followed by a slopy region, both associated with the reduction of molecular sulfur (S_8_) to medium chain polysulfides (Li_2_S_x_, x = 4–6). Afterwards, a second region (II) characterized by a long plateau at lower potential (around 2.1 V vs. Li/Li^+^) appears, resulting from the reduction of lithium polysulfides to form the final solid discharge products (Li_2_S_x_, 1 ≤ x ≤ 2) (Xu et al., 2015).

Figures 1B,C show the evolution of the capacity arising in regions I and II, respectively, for a few selected cycles (1st to 10th and 50th to 60th cycle). The individual capacity of each region is calculated as shown in the inset of Figure 1A. Namely, Region I is delimited between the beginning of discharge until the dip in potential before the low-potential plateau. Region II starts at the dip in potential until the end of discharge. As shown in Figure 1B, the capacity in the first region decrease sharply during the first 10 cycles to stabilize, however, after the 50th cycle. This indicates that, in both cells, less and less elemental sulfur (S_8_) is formed in each charge, until a minimum is reached. In Figure 1C, the evolution of the capacity associated with the second region shows a considerably more peculiar behavior. It is clearly observed that the initial lower capacity of the fresh electrolyte cell with respect to that employing the aged one mostly arose from region II, where the solid discharge products are formed. In fact, while the Li_2_S-CC_A cell delivered a stable capacity upon cycling, the Li_2_S-CC_F cell showed a lower and more rapidly decreasing capacity in this region. This indicates that understanding the effect of the N-rich carbon-coating and the aged electrolyte on this voltage region is vital to a deeper comprehension of the lithium sulfide electrochemistry.

**Figure 2:**
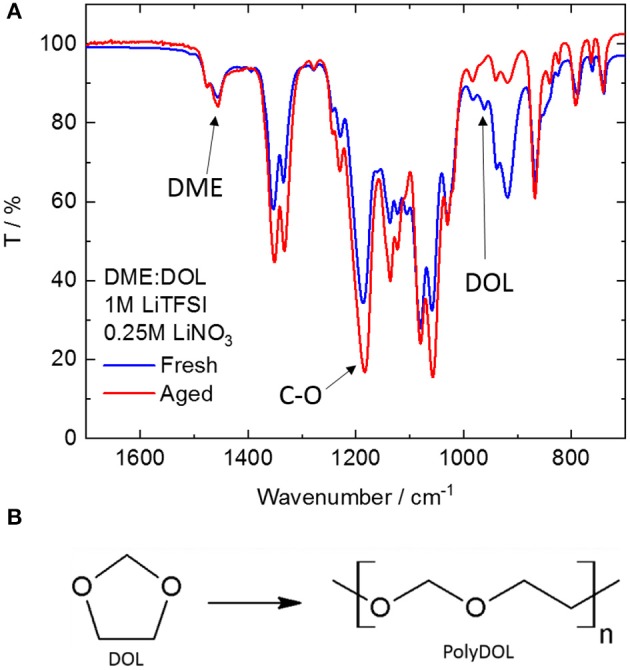
**(A)** ATR-FTIR spectra of freshly prepared (blue line) and aged (red line). DME, DOL-based electrolyte. **(B)** Scheme of the polymerization reaction of DOL.

First of all, the electrolytes were studied by IR spectroscopy. Figure 2 shows the infrared spectrum of a freshly prepared electrolyte (fresh), as well as that of one (with the same composition) after storage for at least 12 months (aged). Clearly, there are significant changes occurring during storage. The band at around 1,450 cm^−1^ due to the –CH_3_ deformation in DME (Bailey, 1985) has a constant intensity before and after aging, showing that DME is stable. On the contrary, the band at 960 cm^−1^ due to the ring-breathing mode of DOL (Yang et al., 2005) shows an appreciable decrease upon storage. In fact, DOL is known to be relatively unstable (Okada et al., 1964), being prone to ring-opening reactions such as isomerization and polymerization (Okada et al., 1964). Isomerization of DOL leads to the formation of ethyl formate. However, no band corresponding to the vibration of the carbonyl group can be detected in the aged electrolyte, thus ruling out this option. On the other hand, polymerization of DOL has been extensively studied and known to happen when a cationic initiator is present (Okada et al., 1964; Berman et al., 1969; Yang et al., 2005), leading to the formation of polydioxolane (polyDOL, Figure 2B), a poly-ether. The formation of polyDOL in the aged electrolyte is proven by the increase in the band at around 1,190 cm^−1^, which is caused by the vibrations of the C-O bonds in linear molecules (Yoshida and Matsuura, 1998). Being a cyclic compound, C-O-C vibrations in DOL involve the whole ring and are thus heavily shifted (Makarewicz and Ha, 2001). The exact cause of this polymerization, in this case, is still unknown. Although DOL is known to be reactive, polymerization only happens in the presence of an appropriate initiator, usually one that can promote chain growth through a cationic mechanism. Analysis of the pure solvent mixture (1:1 DME:DOL) without addition of salt and stored under the same conditions for the same period of time shows no change in its spectrum, such that the culprit is likely the salts added to form the electrolyte (LiNO_3_ and LiTFSI). Li^+^ is known to coordinate the oxygen atoms in ether molecules (Blint, 1995), placing a partial positive charge on it. This weak initiating ability could explain the long storage times necessary to observe the polymerization appreciably.

In summary, the polymerization of DOL occurring upon prolonged storage of the electrolyte appears to be beneficial in Li_2_S-CC_A cell, which shows a higher capacity than the Li_2_S-CC_F cell (Figure 1A) mainly due to a higher and more stable capacity delivered in region II (Figure 1C). Similar results have been already reported in literature (Li et al., 2016). The comparison of electrolytes based on DME:DOL, TEGDME:DOL, and poly(ethylene glycol) dimethyl ether (PEGDME):DOL, for which the chain length increases in the aforementioned order, showed an increase in capacity of up to 2.5 times for the PEGDME:DOL based electrolyte compared to the DME:DOL based one. In order to better clarify the reason for the Li_2_S-CC cell behavior, the EIS response of all electrodes in the discharged states upon cycling was collected.

**Figure 3:**
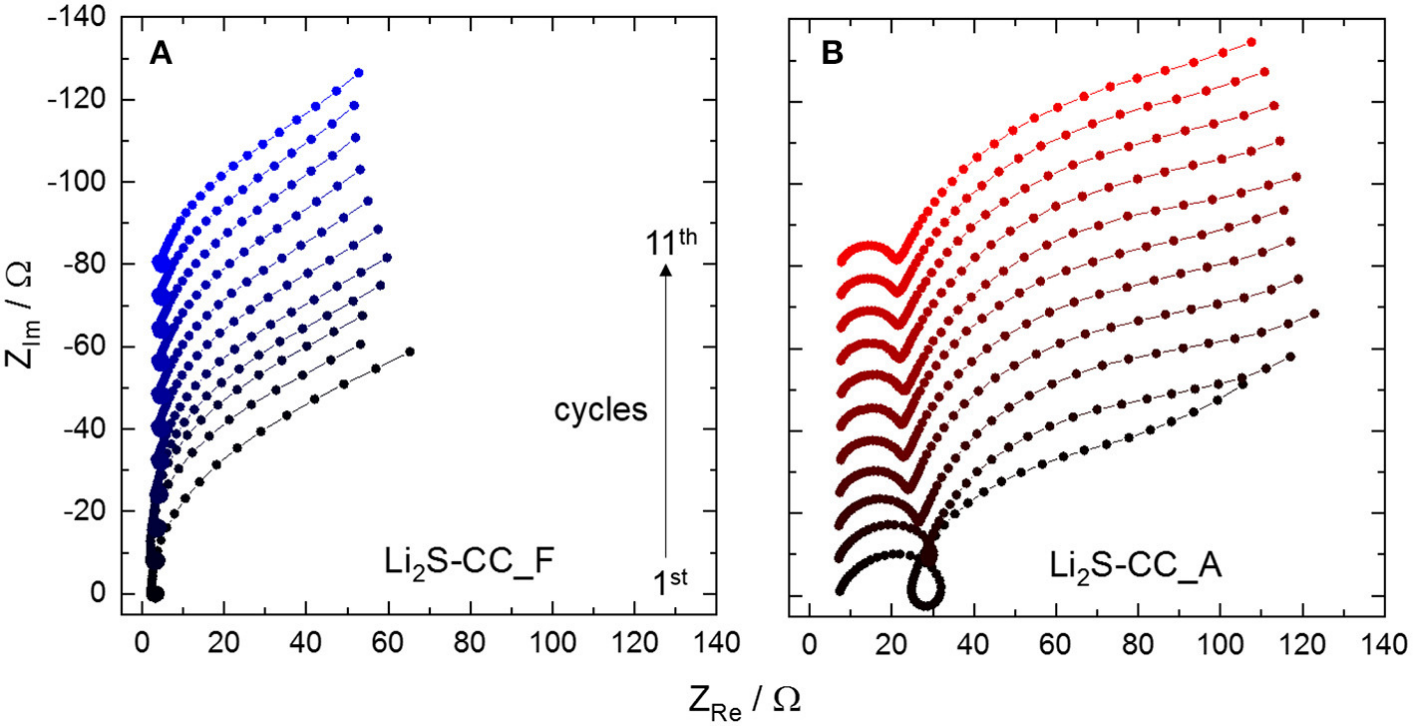
Evolution of the electrochemical impedance spectra from the first to the eleventh cycle of **(A)** Li_2_S-CC_F and **(B)** Li_2_S-CC_A. The baselines of the spectra are shifted upward for clarity. All spectra were taken on 3-electrode cells in the discharged state.

The spectra of Li_2_S-CC_F (Figure 3A) show a small high frequency (HF) semicircle evolving into a pseudo-inductive loop, followed by the beginning of a large, partially depressed, semicircle at low frequencies (LF). The pseudo-inductive loop has been related to intermediate species, i.e., polysulfides, adsorbed on the electrode's surface (Ding et al., 2013; Jeong et al., 2013; Lin et al., 2013; Xie et al., 2017). The depressed LF semicircle is typical of charge-transfer reactions, and corresponds to the reversible conversion of polysulfides to solid products. However, for Li_2_S-CC_A (Figure 3B) some differences are clearly observed, the most relevant being the large HF semicircle before the pseudo-inductive loop. This feature may be associated to the presence of a surface layer on the electrode. The formation of a passivation layer is not unexpected. In fact, during the activation step the cathode is charged up to 4 V vs. Li/Li^+^, while the electrolyte is only stable up to 3.6 V vs. Li/Li^+^ (Yim et al., 2013). To test the effect of the N-rich carbon-coating on the electrode passivation, the anodic stability window of carbon electrodes (Li_2_S-free) was investigated.

**Figure 4:**
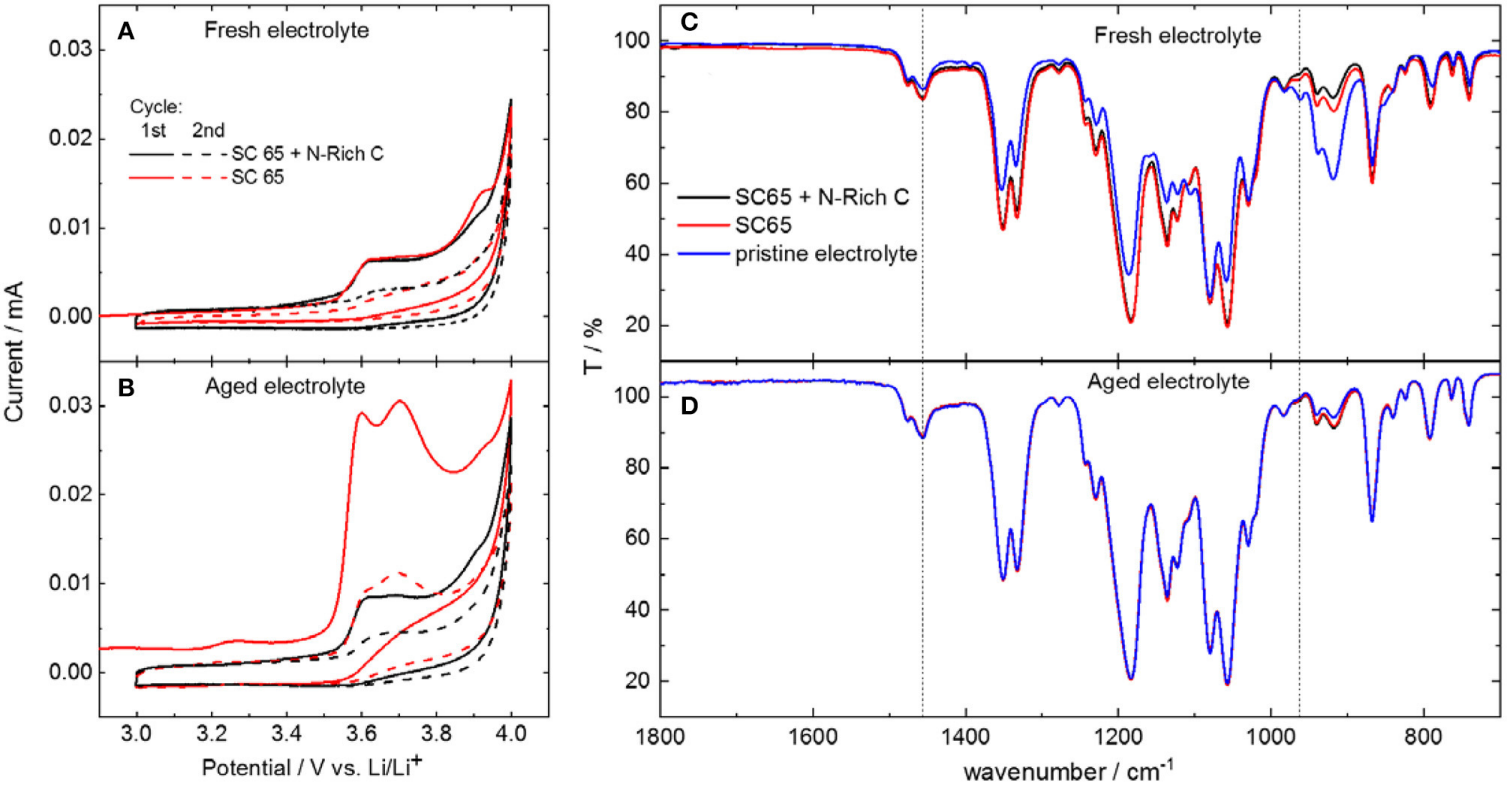
Electrochemical stability window determined by cyclic voltammograms with different carbon-based electrodes, of **(A)** fresh and **(B)** aged electrolytes. IR spectra of **(C)** fresh and **(D)** aged electrolyte extracted from the cell. The spectra of the uncycled electrolyte is also shown for comparison.

Figure 4 shows the anodic stability limit measurements of the electrolytes performed using carbon electrodes containing either the conductive carbon only (labeled as SC 65) or the mixture of conductive carbon and nitrogen-rich carbon (labeled as SC 65 + N-rich C), the latter mimicking carbon coating in Li_2_S-CC electrodes. As seen in Figures 4A,B, the two electrolytes showed oxidation peaks above 3.5–3.6 V vs. Li/Li^+^. For the fresh electrolyte, comparable peaks were observed with both kinds of carbon electrodes in the first cycle. The same occurred in the second cycle, where almost no oxidation current was recorded, indicating that the carbon electrodes underwent a passivation process upon the previous scan. However, the recorded peak current was substantially larger with the aged electrolyte, especially for the electrode solely composed by SuperC 65 and the binder.

The electrolyte was then extracted from such cells and analyzed. The infrared spectra of the electrolytes after cycling (Figure 4C) show the disappearance of the DOL band, indicating its consumption to form the passivation layer on the carbon electrodes. The aged electrolyte (Figure 4D) contains less DOL than the fresh one even before cycling, due to the age-induced polymerization. However, there is only a little, if any, change in the spectra of the recovered aged electrolyte in comparison to the pristine aged electrolyte. Still, the cyclic voltammograms show pretty clearly that the oxidation current of the electrode using only Super C65 is much larger than that of the electrode containing also the N-rich carbon. This is a strong indication that the formation of a passivation layer still occurred, but at a much reduced extent on the electrode containing the N-rich carbon. Though the exact mechanism is not clear yet, it can be proposed that the N-rich carbon layer facilitates the anchoring of the PolyDOL from the aged electrolyte, resulting in the formation of a protective layer on the carbon-coated Li_2_S, which leads to the improved performance of such electrodes in the aged electrolyte.

## Conclusions

In summary, the effect of N-rich carbon-coating of Li_2_S-based cathode was studied. The presence of polyDOL in the aged electrolyte has an unexpected, but appreciable impact on the performance of the Li_2_S electrodes, improving the performance of the N-rich carbon-coated Li_2_S electrodes. The obtained results suggest that PolyDOL can form a stable, hydrophilic passivation layer over the N-rich carbon-coating, which improves the lithium sulfide formation during discharge leading to larger discharge capacities.

## Data Availability Statement

The datasets generated for this study are available on request to the corresponding author.

## Author Contributions

LL performed the experiments. LL and AV analyzed the experimental data. All authors wrote the manuscript and conceived the work.

### Conflict of Interest

The authors declare that the research was conducted in the absence of any commercial or financial relationships that could be construed as a potential conflict of interest.
